# Polyphenolic Compounds, Antioxidant, and Cardioprotective Effects of Pomace Extracts from Fetească Neagră Cultivar

**DOI:** 10.1155/2018/8194721

**Published:** 2018-03-22

**Authors:** Ştefania Silvia Balea, Alina Elena Pârvu, Nastasia Pop, Fernando Zamora Marín, Marcel Pârvu

**Affiliations:** ^1^Department of Horticulture and Landscaping, Faculty of Horticulture, University of Agricultural Sciences and Veterinary Medicine, No. 3-5 Calea Mănăştur Street, Cluj-Napoca 400372, Romania; ^2^Department of Pathophysiology, Faculty of Medicine, “Iuliu Hatieganu” University of Medicine and Pharmacy, No. 4-6 Victor Babeş Street, Cluj-Napoca 400012, Romania; ^3^Department of Biochemistry and Biotechnology, Faculty of Oenology, Rovira i Virgili University, C/Marcel.li Domingo s/n Street, Tarragona 43007, Spain; ^4^Department of Biology, Faculty of Biology and Geology, Babes-Bolyai University, No. 42 Republicii Street, Cluj-Napoca 400015, Romania

## Abstract

Grape pomace is a potential source of natural antioxidant agents. Phenolic compounds and antioxidant and cardioprotective properties of fresh and fermented pomace extracts obtained from *Vitis vinifera* L. red variety Fetească neagră grown in Romania in 2015 were investigated. Grape pomace extracts total phenolic index, total tannins, total anthocyanins, proanthocyanidins, flavan-3-ol monomers, stilbenes, and DPPH free radical scavenger were measured. The effect of a seven-day pretreatment with grape pomace extracts on the isoprenaline-induced infarct-like lesion in rats was assessed by ECG monitoring, serum levels of creatine kinase, aspartate transaminase, and alanine transaminase. Total serum oxidative status, total antioxidant response, oxidative stress index, malondialdehyde, total thiols, and nitric oxide have been also assessed. Higher phenolic content and antioxidant activity were found in fermented pomace extracts when compared to fresh pomace extracts. Pretreatment with grape pomace extracts significantly improved cardiac and oxidative stress parameters. In conclusion, Fetească neagră pomace extracts had a good *in vitro* antioxidant activity due to an important phenolic content. *In vivo*, the extracts had cardioprotective effects against isoprenaline-induced infarct-like lesion by reducing oxidative stress, fresh pomace extracts having a better effect.

## 1. Introduction

Cardiovascular diseases are the first cause of death worldwide. The role of oxidative stress in the pathophysiology of cardiovascular diseases is well documented [1]. Moreover, in cardiovascular pathophysiology, oxidative stress became a promising disease biomarker [1] and an important therapeutic target [2].

Oxidative stress is defined as a misbalance between the oxidants and antioxidants in favour of oxidants that promote damage on biological molecules like lipids, proteins, and DNA. Reactive oxygen species (ROS) and nitrogen reactive species (RNS) have been suggested to increase oxidative stress and to cause diseases such as heart attack, stroke, neurodegenerative diseases, diabetes, and cancer [3]. ROS include the superoxide anion (O_2_^−^), H_2_O_2_, and the hydroxyl radical. Initially, they cause mitochondrial depolarization and then activate a positive feedback loop of ROS-induced ROS release [4]. RNS include NO·and peroxynitrite (ONOO^−^), the coupling product of O_2_^−^ with transient excessive NO. Many studies pointed to the role of peroxynitrite in many cardiovascular diseases by inducing oxidative, nitrative, and nitrosative stress [5].

The failure of antioxidant therapy in human diseases was called the antioxidant paradox, and several explanations have been proposed [6]. One explanation was that oxidative stress was not the cause of specific human diseases but a consequence thereof, another theory was that antioxidants had no tissue- or cell-specific effect, and a third explanation was the lack of an appropriate method to measure oxidative stress. The latest opinion is that antioxidants do not simultaneously inhibit both oxidative stress and inflammation [3].

Many natural products proved to have antioxidant and cardioprotective properties [7] by reducing inflammation and oxidative and nitrative stress [3]. The low incidence of cardiovascular diseases in southern France despite a diet rich in saturated fat, known as the “French Paradox,” was attributed to the antioxidant effect of the regular red wine drinking [8].

A recent point of interest in the winemaking industry is waste management [9]. About 80% of the worldwide grape production is used in winemaking industry. During the winemaking process, after alcoholic fermentation, about 25% of the processed grape weight remains as organic solid waste, the grape pomace (GP). It consists mainly of skin residues, broken cells with pulp remains, stalks and seeds. Large quantities of GP are produced annually, and it is used mainly for animal food, organic fertilizers, and ethanol production [10]. In Romania, GP is disposed as a waste. Grapes, GP, and wines from red varieties of *Vitis vinifera* L. are important sources of natural antioxidants, because of their high concentration of different phenolic compounds [11]. About 70% of the phenolic compounds were reported to remain in the pomace [12]. The majority of grape polyphenols come from the skin and seeds. They comprise two main classes: flavonoids (anthocyanins, flavonols, flavan-3-ols, flavones, and chalcones) and nonflavonoids (phenolic acids, stilbenes, tannins, coumarins, and neolignans) [10].

Moreover, in the case of the polyphenolic compounds, it has been found that they have antioxidant properties but they may also act as prooxidants because they may induce free radical production [13]. Another opinion is that the prooxidant effect of grape pomace extract might be beneficial because it triggers the preconditioning mechanisms [14].

Considering all these previous findings, in this paper, we present the results of performing a phytochemical analysis and investigation of the antioxidant and cardioprotective effects of GP obtained from *Vitis vinifera* L. red variety Fetească neagră (FN) grown in Romania. To the best of our knowledge, there is no research published on the antioxidant and cardioprotective effects of Fetească neagră GP.

## 2. Materials and Methods

### 2.1. Chemicals and Reagents

All solvents were of HPLC quality, and all chemicals were of analytical grade (>99%). Methanol, ethanol (96.5%), deionised water, acetonitrile, formic acid, absolute ethanol, and hydrochloric acid (37%) were purchased from Panreac (Barcelona, Spain). The commercial standards transresveratrol, transpiceid, and (−)-epicatechin were bought from Phytolab (Vestenbergsgreuth, Germany) and Extrasynthese (Genay, France), respectively. The transisomers of resveratrol and piceid (resveratrol-3-glucoside) were transformed into their respective cis isomers by UV irradiation (366 nm light for 5 min in quartz vials) of 25% MeOH solutions of the transisomers. Trolox (6-hydroxy-2,5,7,8-tetramethylchroman-2-carboxylic acid), N-(1-Naphthyl)ethylenediamine dihydrochloride (NEDD), xylenol orange [o-cresosulfonphthalein-3,3-bis (sodium methyliminodiacetate)], ortho dianisidine, vanadium (III) chloride (VCl3), hydrogen peroxide (H_2_O_2_), methanol, diethyl ether sulphanilamide (SULF) and ferrous ammonium sulphate, thiobarbituric acid, trichloroacetic acid (TCA), ethylenediaminetetraacetic acid, sodium dodecylsulphate, butylated hydroxytoluene, 1,1,3,3-tetraethoxypropane, 5,5′-dithiobis (2-nitrobenzoic acid) (DTNB), and glutathione (GSH) were purchased from Sigma-Aldrich (Germany) and Merck (Germany). All chemicals were of analysis grade.

### 2.2. Grape Sample

The grape *Vitis vinifera* (L.) var. Fetească neagră (clone 762, *Vitis* L., port graft: S.O.4, Austria) planted in 2006, from Mureş county, Mica parish, part of Târnavelor Plateau (46°21′44.5′′N and 24°23′55.7^″^E; 330–350 m above sea level), was used in our studies. Grapes were harvested manually at full maturity level, during the 2015 vintage. The GP samples were collected in two winemaking stages: one was supplied immediately after pressing the grapes, the fresh unfermented GP (FNFs), and the other was supplied after 20 days of fermentation at 20°C and must separation, the fermented GP (FNFr). The samples were stored in vacuum bags at −22°C prior to the analysis and were used in the experiments.

### 2.3. Sample Preparation for Phytochemical Analysis

The GP was submitted to the freeze-drying process and then ground with a domestic blender (Sinbo, model number SCM 2923). The extracts were prepared by adding 50 mL of ethanol 50% and 1 mL of metabisulphite (MBS) 5.25% to 5 g of GP sample. All extracts were placed in a water bath at 50°C for 7 days and stirred daily. Prior to each analysis, the extracts were centrifuged and the supernatants were further used. All samples were processed in 3 repetitions.

### 2.4. Total Phenolic Index, Total Tannins, and Total Anthocyanin Determination

The total phenolic index (TPI) was determined by measuring 280 nm absorbance of a 1 : 200 dilution of GP extracts [15]. The condensed tannin concentration (TC) was estimated by precipitation with methyl cellulose [16]. The absorbance was read at 280 nm, and TC was calculated as the difference between the total polyphenols and the quantity of tannins precipitated by methyl cellulose. Aqueous (−)-epicatechin solutions were used to establish a standard curve, and TPI and TC were expressed as epicatechin mg/g d.w. The total anthocyanin content (TAC) was determined spectrophotometrically using the method described by [17]. The absorbance was read at 520 nm. A standard curve was made using malvidin-3-O-glucoside chloride, and results were expressed as malvidin-3-O-glucoside mg/g d.w. Spectrophotometric measurements for TPI, TT, and TAC were performed with a Helios Alpha UV-Vis spectrophotometer, Thermo Fisher Scientific Inc., Waltham, MA, USA.

### 2.5. HPLC-DAD Determination of Proanthocyanidins and Flavan-3-ol Monomers

The proanthocyanidins of the GP were extracted and analyzed by acid depolymerization in the presence of an excess of phloroglucinol [18]. The products of the reaction were separated by RP-HPLC-DAD. The proanthocyanidins were analyzed with an Agilent 1200 Series HPLC equipped with a G1362A refractive index detector (RID), a G1315D DAD, a G1311A quaternary pump, a G1316A column oven, and a G1329A autosampler (Agilent Technologies, Santa Clara, CA, USA). The chromatographic system was managed by an Agilent Chem Station (version B.01.03) data processing station. The number of terminal subunits was considered to be the difference between the total monomers measured in normal conditions (with phloroglucinol) and thus obtained when the analysis was performed without phloroglucinol addition. The number of extension subunits was considered as the addition of all the phloroglucinol adducts. Because acid catalysis with phloroglucinol is not completely efficient, the real yield of the reaction was measured using a pure B2 proanthocyanidin dimer [(−)-epicatechin-(4 → 8)-(−)-epicatechin]. This output was used to calculate the total proanthocyanidin concentration from extracts. The mean degree of polymerization (mDP) was calculated by adding terminal and extension subunits (in moles) and dividing by the terminal subunits. The percentage of prodelphinidins (PD%) was computed by dividing the total (−)-epigallocatechin units by the total monomeric units and converting the result to a %. Similarly, the percentage of galloylation (G%) was computed by dividing the total (−)-epicatechin-3-gallate units by the total monomeric units and converting the result to a %. In order to quantify the flavan-3-ol monomers, (+)-catechin, (−)-epicatechin, and (−)-epicatechin-3-O-gallate in GP, the assay was also carried out without the addition of phloroglucinol and the retention times were compared with those of pure compounds. All analyses were performed in three repetitions.

### 2.6. HPLC-DAD-ESI-MS/MS Determination of Stilbenes

HPLC identification of GP stilbenes was performed using an Agilent 1200 series system equipped with DAD (Agilent, Germany) and coupled to an AB Sciex 3200 Q TRAP (Applied Biosystems) electrospray ionization mass spectrometry system (ESI-MS/MS) [19]. The chromatographic conditions used were conditions that had been previously reported [20]. The chromatographic system was managed by an Agilent Chem Station (version B.01.03) data process in G station. The mass spectral data was processed with the Analyst MDS software (Applied Bio-systems, version 1.5) [21].

### 2.7. DPPH Radical Scavenging Activity

The antioxidant capacities of the GP samples were measured in terms of their radical scavenging activity (RSA), using the DPPH method [22, 23]. Briefly, a 3 ml aliquot of the extract solution was added to 1 mL of methanol solution of DPPH 0.1 mM. The mixture was then homogenized and kept in the dark at room temperature for 30 min prior to analysis. The absorbance was measured at 517 nm against a blank. The following equation was used to determine the percentage of the radical scavenging activity of each extract: RSA (%) = [1 − (Asample − Ablank)/Acontrol] × 100, where Acontrol is the absorbance of DPPH radical + methanol, Asample is the absorbance of DPPH radical + sample, and Ablank is the absorbance of methanol + sample. The IC_50_ (half maximal inhibitory concentration) was calculated graphically, using a calibration curve, in the linear range by plotting the extract concentration versus the corresponding scavenging effect (RSA%), over 30 min. Trolox was used as a positive antioxidant control. The percentage of DPPH consumption was converted to trolox equivalents (TE) using a calibration curve (R2 = 0.985) of Trolox standard solutions (0.5–5 *μ*g/mL). An IC_50_< 50 *μ*g TE/ml is a very good antioxidant activity; an IC_50_ of 50–100 *μ*g TE/ml is a good antioxidant activity; an IC_50_ of 100–200 *μ*g TE/ml is a week antioxidant activity; an IC_50_> 200 *μ*g TE/ml means no antioxidant activity. Assay was performed in triplicate.

### 2.8. Plant Extract Preparation for In Vivo Study

Fetească neagră fresh GP extract (FNFs) and Fetească neagră-fermented GP extract (FNFr) were obtained with 70% ethanol (Merck, Bucuresti, Romania) by a modified Squibb repercolation method (1/1 g/ml) [24].

### 2.9. Animals

The experiments were carried out on male albino Wistar rats, weighing 200–250 g, that were bred in the Animal Facility of the Iuliu Hațieganu University of Medicine and Pharmacy. The animals were housed in standard polypropylene cages (five per cage) under controlled conditions (12 h light/dark cycle, at an average temperature of 21-22°C) and with ad libitum access to standard pellet diet (Cantacuzino Institute, Bucharest, Romania) and water.

Experimental protocols have been approved by the Ethics Committee (nr. 26/16.12.2015) of the Faculty of Veterinary Medicine, University of Agricultural Sciences and Veterinary Medicine from Cluj-Napoca and the Ethics Committee of the Iuliu Hațieganu University of Medicine and Pharmacy. The experiments were performed in triplicate. All experimental groups began with 3 days of acclimatization to the housing facility, and animals were used only once. At the end of the experiments under anaesthesia using a combination of ketamine (60 mg/kg bw) and xylazine (15 mg/kg bw) [25], blood was withdrawn by retroorbital puncture, serum was separated and stored at −80°C until use, and then animals were killed by cervical dislocation.

### 2.10. Experimental Myocardial Ischemia

The animals were divided into 4 groups (*n* = 5). The negative control group (CONTROL) and the isoprenaline-induced (ISO) myocardial infarction group received 0.9% saline (1 ml/day p.o.) for 7 days. In FNFs and FNFr groups, each extract was administrated orally by gavage (1 mL/day p.o.) for 7 days. In day 8 and day 9, rats were injected with ISO dissolved in normal saline (150 mg/kg, s.c.) at an interval of 24 h to induce experimental MI [26], excepting CONTROL animals. ECG was recorded in days one, 7, and 10. In day 10, after ECG registration, blood samples were collected for estimation of cardiac markers and oxidative stress markers.

### 2.11. Electrocardiography

The overnight-fasted rats were anaesthetized with ketamine (80 mg/kg, i.p.) and xylazine (8 mg/kg, i.p.). At 15 min after anesthesia, animals were placed in the supine position on a board, electrodes were bound on the paw pads of each rat, and ECG was recorded from the limb lead at position II (right forelimb to left hind limb) with a Biopac MP150 system. The ECG apparatus was calibrated at 1 mV/1 cm with a speed of 50 mm/s.

Analysis of ECG waves was done to calculate heart rate (beats/min), RR intervals (msec), QT interval (msec), and ST segment changes (mV). QT interval was measured from the beginning of QRS complex to the end of T wave, and it was calculated in msec. Corrected QT interval (QTc), which is used to rectify the influence of the heart rate on QT interval, according to Bazett formula, is equal to QT interval divided by the square root of RR interval and was also calculated [27].

### 2.12. Cardiac Marker Enzymes

The creatine kinase-MB (CK-MB), aspartate transaminase (AST), and alanine transaminase (ALT) activities were measured by using commercial kits.

### 2.13. In Vivo Antioxidant Effect Evaluation

The total oxidative status (TOS) of the serum was measured using a colorimetric assay [28]. Assay measurements were standardized using hydrogen peroxide (H_2_O_2_) as the oxidative species, and the assay results are expressed in *μ*mol H_2_O_2_ equiv/L.

The total antioxidant response (TAR) was measured in serum using a colorimetric assay [29]. This assay is calibrated using trolox, and results are expressed as mmol trolox equiv/L.

The ratio of the TOS to the TAR represents the oxidative stress index (OSI), an indicator of the degree of oxidative stress: OSI (arbitrary unit) = TOS (mol H_2_O_2_ equiv/L)/TAR (mmol trolox equiv/L) [30].

Malondialdehyde (MDA) was assessed as a lipid peroxidation marker, using thiobarbituric acid, as previously described [31]. The absorbance of the supernatant was measured at 532 nm. A standard curve was generated with a 1,1,3,3-tetraethoxypropane standard (0.3–10 nmol/mL). Serum MDA concentration was expressed as nmol/mL of serum.

Total thiols (SH) were estimated using Ellman's reagent [32]. Supernatant absorbance was measured at 412 nm. To create a standard curve, solutions of glutathione (GSH) concentration, ranging from 0.25 to 2 mM GSH, were used. Serum SH concentration was expressed as mmol GSH/mL.

The Griess reaction was used to indirectly determine NO synthesis (NOx). First serum proteins were removed by extraction with a 3 : 1 (*v* : *v*) solution of methanol/diethyl ether [33]. The sample absorbance was read at 540 nm. The concentration of serum NOx was determined using a sodium nitrite-based curve and expressed as nitrite *μ*mol/L [34].

All serum spectrophotometric measurements were performed using a Jasco V-530 UV-Vis spectrophotometer (Jasco International Co. Ltd., Tokyo, Japan).

### 2.14. Statistical Analyses

Results were expressed as means and standard deviations and were analyzed statistically using the SPSS (version 20) software. Data were compared by one-way ANOVA and post hoc Bonferroni-Holm test. Pearson's correlation coefficient (*r*) was used to evaluate relationships between parameters of the same group. The level of significance was set at *p* < 0.05.

## 3. Results

### 3.1. Total Phenolic Index, Total Tannins, and Total Anthocyanin Content

The TPI, TAC, and TC obtained from Fetească neagră GP are shown in Table 1. In the present study, FNFr pomace had significantly higher polyphenol extraction (161.58 ± 3.42 mg catechin equivalent/g d.w.) than FNFs (114.71 ± 15.86 mg catechin equivalent/g d.w.) (*p* < 0.05). TC in GP ranged from 63.15 ± 7.12 mg epicatechin/g d.w. in FNFs to 113.98 ± 4.39 mg epicatechin/g d.w. in FnFr, the difference being very significant (*p* < 0.001). Higher TAC was found in FNFs (184.84 ± 17.13 mg malvidin-3-O-glucoside/g d.w.) than in FNFr (47.67 ± 5.0 mg malvidin-3-*O*-glucoside/g d.w.). Between TAC of FNFs and FNFr, the difference was highly significant (*p* < 0.001).

### 3.2. HPLC-DAD Determination of Proanthocyanidins and Flavan-3-ol Monomers

The results of analyzing GP proanthocyanidins obtained by acid depolymerization in the presence of excess phloroglucinol are shown in Table 2. The total proanthocyanidin concentration is higher in FNFr than in FNFs (*p* < 0.05). As shown in Table 1, the mDP of FNFr and FNFs were not significantly different (*p* > 0.05), PD% in FNFs was significantly bigger than that in FNFr (*p* < 0.01), and G% in FNFr was higher than that in FNFs but not significant (*p* > 0.05).

The flavan-3-ol monomeric compositions from the whole Fetească neagră GP were described in a detailed form in Table 1. With respect to the total flavan-3-ol monomer concentration, we found that it was higher in the FNFr sample than in the FNFs sample (*p* < 0.01). The analysis of individual flavan-3-ol monomers showed that the difference in total monomers concentrations was due to the higher content of (+)-catechin and (−)-epicatechin in the FNFr samples (*p* < 0.01). The epicatechin-3-O-gallate concentration was not significantly different between the FNFr and FNFs samples (*p* > 0.05).

### 3.3. HPLC-DAD-ESI-MS/MS Determination of Stilbenes

The transresveratrol was in a higher amount in the FNFs (*p* < 0.01) than in the FNFr and cis-resveratrol in a higher amount in the FNFr (*p* < 0.01) than in the FNFs. In both FNFs and FNFr samples, the concentration of cis-resveratrol was higher than the concentration of transresveratrol. Piceid was the most abundant stilbenes in GP from both FNFs and FNFr, and both isomers cis- and transpiceid were in a greater quantity in the FNFs samples as compared to the FNFr samples (*p* < 0.001) (Table 1).

### 3.4. Radical Scavenging Activity toward DPPH

**Figure 1:**
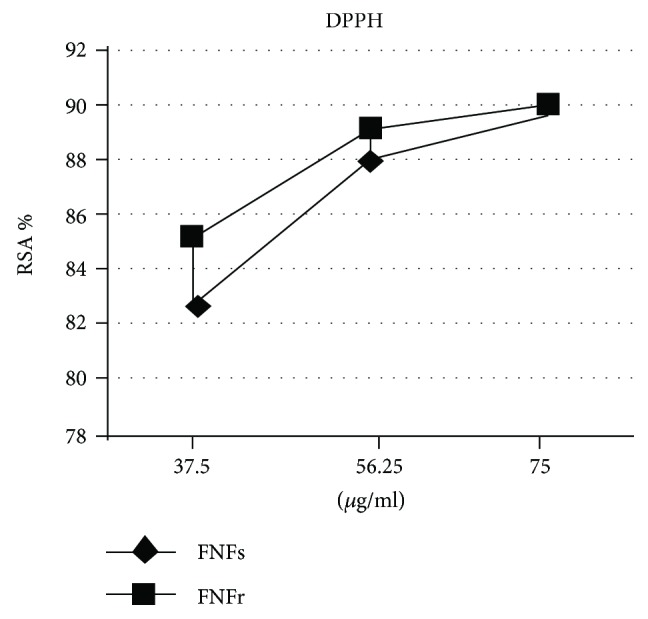
Fetească neagră grape pomace radical scavenging activity toward DPPH. Fs: fresh, Fr: fermented, FN: Fetească neagră, RSA: radical scavenging activity.

The ethanol extracts of FNFs and FNFr showed very good RSA by having an increased absorbance with increased concentration at 517 nm (Figure 1). Compared to trolox (IC_50_ = 11.18 *μ*g/ml), FNFr (IC_50_ = 9.95 *μ*g TE/ml) exhibits a better antioxidant activity than FNFs (IC_50_ = 36.99 *μ*g TE/ml), and there was a statistically significant difference between these extracts (*p* < 0.001). For FNFs, DPPH test results exhibited significant, positive correlations with TC, TAC, flavan-3-ol monomers, and stilbene results (*r* = 0.80–0.99). The FNFr DPPH test correlated with proanthocyanidins, PD, mDP, and cis-stilbenes (*r* = 0.76–0.90).

### 3.5. Effect of Fetească Neagră Grape Pomace Extracts on ECG Parameters

**Figure 2:**
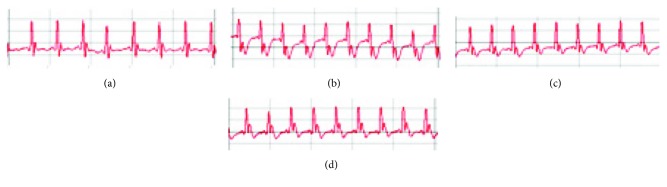
Chart records showing the effect of Fetească neagră grape pomace fresh extract (FNFs) and Fetească neagră grape pomace-fermented extract (FNFr) pretreatments on ECG tracings: (a) normal control, (b) isoproterenol (ISO), (c) FNFs, and (d) FNFr. The electrocardiogram was recorded from limb leads II in each group. The sensitivity was 1 mV, and the chart speed was 50 mm/sec.

ECG recording from days one and 7 had no significant changes in all groups. The ECG patterns of normal and experimental group rats in day 10 are shown in Figure 2 and Table 2. Isoprenaline injection was seen to induce significant alterations in ECG patterns such as decreased heart rate, increased RR, QT and QTc intervals, and ST segment depression coupled with marked T wave inversion that reflect isoprenaline-induced infarct-like lesion. Pretreatment with FNFs produced protective effects on ECG by reducing RR, QT, and QTc increase, by increasing the HR and reducing ST depression as compared to ISO-treated group (Table 2). Pretreatment with FNFr reduced just RR interval, increased the HR, and reduced ST depression as compared to ISO-treated group.

### 3.6. Effect of Fetească Neagră Grape Pomace Extracts on Cardiac Marker Enzymes

The cardioprotective effects of Fetească neagră GP extracts revealed by the serum cardiac marker enzyme AST, ALT, and CK-MB levels are summarized in Table 3. Serum levels of cardiac marker enzymes, AST, ALT, and CK-MB, increased significantly in ISO-treated rats (*p* < 0.001). FNFs and FNFr extract pretreatment produced protective effects by reducing significantly AST, ALT, and CK-MB levels. On AST, FNFs effect (*p* < 0.01) was better than that of FNFr (*p* < 0.05). ALT was significantly reduced by both pretreatments, FNFs and FNFr (*p* < 0.01), and the results were almost similar. FNFr lowered CK-MB more than FNFs did (*p* < 0.001). The cardioprotective effects of FNFs and FNFr on cardiac enzymes were correlated with ST segment changes.

### 3.7. In Vivo Antioxidant Effect of Fetească Neagră Grape Pomace Extracts

Oxidative stress tests are summarized in Table 4. Significant increases in serum OSI (*p* < 0.001) and TOS (*p* < 0.001) with concomitant decrease in TAR (*p* < 0.01) were seen in the ISO-induced MI rats. In contrast, individual preadministration of FNFs and FNFr resulted in a significant decrease in the levels of TOS (*p* < 0.001) and OSI (*p* < 0.001) as well as significant increases in TAR (*p* < 0.01). However, when FNFs and FNFr groups were compared to each other, the ANOVA analysis revealed that the decrease in TOS and OSI levels and the increase in TAR were not significantly different. Furthermore, OSI, TOS, and TAR changes were correlated. ISO-induced ischemia was associated with a significant elevation of MDA (*p* < 0.01), and FNFs and FNFr pretreatments reduced MDA production (*p* < 0.05). FNFs and FNFr were equally efficient on MDA (*p* > 0.05). SH was reduced by the ISO-induced ischemia (*p* < 0.001), and pretreatment with FNFs and FNFr extracts had no important effect on SH (*p* > 0.05). Serum NOx was significantly elevated in the ISO group (*p* < 0.001) as compared to the control rats. Pretreatment with FNFs and FNFr extracts induced no significant reduction of nitric oxide production (*p* > 0.05). There was no significant statistical difference between FNFs and FNFr extracts in terms of NOx production (*p* > 0.05).

## 4. Discussion

In the present study, polyphenolic composition and antioxidant and cardioprotector effects of fresh and fermented FN GP were analysed. The FNFs and FNFr demonstrated to be rich sources of phenolic compounds. It is already known that phenolic compounds represent one of the largest and important groups of natural plant products, having physiological properties such as antiallergenic, anti-inflammatory, antimicrobial, antioxidant, antithrombotic, cardioprotective, and vasodilatory effects [35]. There is a growing consensus that a combination of antioxidants, rather than single entities, may be more effective over the long term [36].

As in other studies, FNFs and FNFr phenolic contents were high [37]. The differences between GP phenolic contents are related to the grape variety, grape ripeness, environmental factors, and technological procedures used during winemaking. In wine, the total phenolic content was higher when the maceration time was longer [10]. In our samples, the higher TPI in the fermented compared to the fresh FN GP may be attributed to the maceration process.

Tannins are localized mainly in the skin and seeds of grapes. Tannin content in FN GP followed the same trend as TPI, FNFr having a higher tannin yield than FNFs. These results were in accordance with reports that found tannin extraction efficiency from unfermented grape skins in a hydroalcoholic solution lower than 38% [38]. The cell wall degradation during fermentation enhances tannin recovery [39]. When samples were compared with other studies from six red wine cultivars from France, after vinification, GP total tannin contents were comparable [40].

Between different cultivars, there are differences of TAC [41]. Moreover, anthocyanins accumulate gradually in the grape skin because their synthesis begins during veraison and continues during the ripening process [42], and after GP maceration, the anthocyanin concentration decreases [43]. It was appreciated that 77% of the anthocyanins are released in this process, resulting in a GP with a low content of these pigments. Furthermore, within the anthocyanin family, due to structural differences, they have different extractabilities [38]. Additionally, the formation of polymeric pigments decreases the amount of free anthocyanins [43]. Furthermore, anthocyanins may be degraded and/or absorbed by yeasts and the tank surface [42]. All these processes explain why TAC diminished in the FNFr sample. The result was in accordance with other studies [44].

The total proanthocyanidin of wines is affected by maturity and maceration. Proanthocyanidin concentration is higher at veraison, decreases until ripeness, and then remains relatively constant. Then, a longer maceration increases the proanthocyanidin content [42]. By putting together the two changes, we may explain why there were small differences between the FNFs and FNFr samples, proanthocyanidin concentrations, namely the maturity-induced lowering being masked by the maceration-induced increase.

Proanthocyanidin structures differ according to their origin. In wines, the proanthocyanidin mean degree of polymerization (mDP) and the percentage of PD increased with maturity, whereas the G% decreased. Furthermore, maceration length also has a significant effect on the proanthocyanidin mDP of wines. Throughout maceration time, the proportion of PD tends to decrease [42], but the G% increases because skin (−)-epigallocatechin is released more quickly [42]. The results obtained for FN GP were in accordance with the observations on wine, mDP, and PD% being higher in FNFs and G% being higher in FNFr.

Flavan-3-ols are extracted from grapes during the vinification process, but not completely, remaining in great amounts in the winemaking residue. Like in other studies, analysing red GP flavan-3-ol monomers [45], in FN GP extracts epicatechin-3-O-gallate were in lower concentration than (+)-catechin and (−)-epicatechin. The higher (+)-catechin and (−)-epicatechin concentrations in FNFr compared to FNFs were attributed to the better extraction of the flavan-3-ols from the skins during the maceration.

Stilbenes are among the main polyphenols involved in the health protection effects of drinking wine. The main grape stilbenes are trans- and cis-resveratrol, glycosylated derivatives of resveratrol *trans*- and *cis*-piceid, piceatannol, and resveratrol dimers (viniferins) [46]. Several *in vitro* studies have shown that resveratrol has antioxidant, anti-inflammatory, cardioprotective, and platelet antiaggregant activities, and glycosylated stilbenes have antioxidant effects [47]. Significant stilbene concentration differences were also observed between GP and skin [48] and between GP, wine, must, and grapes [49]. In FNFs and FNFr, resveratrol and piceid were found in the form of *trans*- and *cis*-isomers as previously described for other red GP [50, 51]. Total stilbene content from FNFr agreed with other studies [52], but FNFs stilbenes were above the values found in other fresh GP. The considerably higher cis-resveratrol in FNFr may be explained by the observation that during vinification transresveratrol is isomerized to the *cis*-form [53]. Because piceid has greater bioavailability than resveratrol [54] and may be hydrolyzed releasing resveratrol [55], high piceid concentration from FNFs and FNFr may represent an important health benefit.

An actual trend is to reduce oxidative stress by using herbal supplements or functional foods rich in natural antioxidants. FNFs and FNFr *in vitro* antioxidant activities were evaluated with the most frequently used method, the DPPH test [36]. The high content of phenolic compounds from the FNFs and FNFr samples explained the good antioxidant activity evaluated by DPPH test. FNFr had a better DPPH IC_50_ than FNFs. This observation is consistent with other studies indicating that the fermentation process increases polyphenol releases from the pomace cells [56]. As phenolic compounds are the most important grape secondary metabolites with antioxidant properties, a high and significant correlation between FNFs and FNFr *in vitro* antioxidant activities and TPI was found. The different structure of each phenolic compound explains their specific efficiency to scavenge different free radicals [10].

The antioxidant capacity of plant extracts cannot be evaluated by using a single test. Studies comparing the *in vitro* and *in vivo* antioxidant effects of a plant extract have shown that some extracts exhibit antioxidant activity both *in vitro* and *in vivo*, but for other extracts, the *in vitro* antioxidant activity does not apply to *in vivo* models [14]. Due to those observations, after demonstrating the promising antioxidant capacity by DPPH test, the FN GP extracts were examined in terms of their *in vivo* cardioprotective and antioxidant effects in experimental rat ISO-induced MI [26].

When injected in rats, isoprenaline undergoes autooxidation and induces myocardial cell membrane injury [57]. ISO induced ECG infarct-like changes, such as heart rate reduction, RR, QT and QTc interval increase, T wave inversion, and ST segment depression. It was considered that such alterations could be due to the consecutive loss of cell membrane potential in the injured myocardium as a result of oxidative stress. Pretreatments with FN GP extracts had cardioprotective effects because they reduced ISO-induced ECG ischemic changes. According to ECG effects, FNFs was a better cardioprotective treatment than FNFr.

Myocardial injury leads to loss of cell membrane structural integrity, with increased permeability and enzyme leakage. The degree of AST, ALT, and CK-MB increase in serum reflects the severity of myocardial injury [58]. Like in other reports, the increase of AST, ALT, and CK-MB correlated well with ST depression in ISO-treated group [57]. FNFs and FNFr pretreatments were cardioprotective by causing a correlated reduction of cardiac enzyme markers and ECG improvements.

The myocardium is vulnerable to oxidative damage because it has limited antioxidant defence possibilities [59]. In MI, the leading cause of myocardial injury is the imbalance between oxidant and antioxidant defences [60]. It was also proved that oxidative stress is coupled with an inflammatory response [61]. Because ROS can freely cross intracellular membranes, plasma can be significantly exposed to ROS leading to systemic consequences [62].

In the present study, oxidative stress was evaluated systemically by measuring serum oxidative stress markers. As a result, an increased oxidative stress, characterized by the TOS, OSI, and MDA elevation associated with TAR lowering, has been found. Both FNFs and FNFr had cardioprotective effects by reducing oxidative stress markers, but FNFs had better *in vivo* activity. The antioxidant activity of plant extracts is the result of the combined effects of many different compounds. It was demonstrated that the antioxidant *in vitro* activity does not always apply to *in vivo* models. Moreover, it was proved that *in vitro* and *in vivo* antioxidant effects may not correspond because polyphenols may also act as prooxidants via Fenton reaction [14].

Nitric oxide (NO) is an important signalling messenger known to play important roles in many physiological and pathological conditions. Endogenous NO is generated from L-arginine by three major nitric oxide synthases: endothelial, neural, and inducible nitric oxide synthases (iNOS). NO plays an important role in host defence and homeostasis when generated at a low level for a brief period of time, whereas the high level or prolonged induction of NO may contribute to a variety of pathological phenomena associated with inflammatory processes [63]. In the present study, ISO-induced myocardial ischemia was associated with an increase of NOx. Preconditioning myocardium with resveratrol has been shown to be related to the stimulation of iNOS [64] with increased NO synthesis acting as an antioxidant [65]. FNFs and FNFr pretreatments had no important effect on NOx concentration after ISO administration. Although enhanced peroxynitrite formation contributes to the pathophysiology of cardiovascular diseases, it was demonstrated that nanomolar concentrations of peroxynitrite inhibit leukocyte-endothelial cell interaction, which improves postischemic myocardial function [66]. Moreover, it was reported that peroxynitrite plays a role in triggering ischemic preconditioning and ischemic postconditioning [67].

## 5. Conclusion

In conclusion, fresh or fermented Fetească neagră grape pomace extract had a good *in vitro* antioxidant activity due to an important phenolic content. *In vivo* Fetească neagră grape pomace extracts had cardioprotective effects against ISO-induced myocardial ischemia by reducing oxidative stress. The *in vitro* and *in vivo* antioxidant effects were different, respectively, fermented Fetească neagră grape pomace had a better *in vitro* antioxidant activity, and fresh Fetească neagră grape pomace had a stronger *in vivo* antioxidant activity. Overall, the findings presented in this study suggest for the first time that Fetească neagră grape pomace extract pretreatment may be an option for heart preconditioning. Further studies are required to investigate the cardioprotective effects of Fetească neagră grape pomace extracts over a longer period.

## Figures and Tables

**Table 1:** Phytochemical analysis of grape pomace from cv. Fetească neagră 2015.

GP	TPI (mg catechin equivalent/g d.w.)		TC (mg epicatechin/g d.w.)	TAC (malvidin-3-O-glucoside mg/g d.w.)
FNFs	114.71 ± 15.86		63.15 ± 7.12	184.84 ± 17.13^∗∗∗^
FNFr	161.58 ± 3.42^∗∗^		113.98 ± 4.39^∗∗∗^	47.67 ± 5.0

	Proanthocyanidins (mg/g d.w.)	mDP	PD%	G%

FNFs	25.57 ± 3.82	6.29 ± 0.16^n.s.^	8.18 ± 0.49^∗∗^	19.93 ± 0.41
FNFr	28.71 ± 9.78^∗^	5.64 ± 1.33	3.91 ± 1.12	21.69 ± 1.50^n.s.^

	∑ flavan-3-ol monomers (mg/g d.w.)	(+)-catechin (mg/g d.w.)	(−)-epicatechin (mg/g d.w.)	epicatechin-3-O-gallate (mg/g d.w.)

FNFs	2.05 ± 0.21	1.12 ± 0.10	0.87 ± 0.09	0.06 ± 0.01
FNFr	4.71 ± 0.17^∗∗^	2.59 ± 0.01^∗∗^	2.05 ± 0.16^∗∗^	0.07 ± 0.00^n.s.^

	Transresveratrol (*μ*g/g d.w.)	cis-resveratrol (*μ*g/g d.w.)	Transpiceid (*μ*g/g d.w.)	cis-Piceid (*μ*g/g d.w.)

FNFs	3.22 ± 1.91^∗∗^	5.03 ± 1.09	53.54 ± 10.53^∗∗^	33.04 ± 8.14^∗∗^
FNFr	0.58 ± 1.01	12.27 ± 3.07^∗∗^	14.31 ± 2.25	7.67 ± 1.99

Values are expressed as average ± standard deviation (*n* = 3) ^n.s.^*p* > 0.05, ^∗^*p* < 0.05, ^∗∗^*p* < 0.01, and ^∗∗∗^*p* < 0.001. GP: grape pomace; Fs: fresh; Fr: fermented; FN: Fetească neagră; TPI: total phenolic index; TC: tannins concentration; TAC: total anthocyanin content; mDP: mean degree of polymerization; PD: prodelphinidins; G%: galloylation.

**Table 2:** RR interval, QT interval, QTc, heart rate, and ST segment of rats, day 10 ECG.

	RR (ms)	QT (ms)	QTc (ms)	HR (bpm)	ST (mV)
CONTROL	238 ± 22.80	80 ± 14.14	198 ± 36.33	213.31 ± 22.18	00
ISO	256^∗^ ± 21.90	136^∗∗^ ± 16.73	274^∗∗^ ± 43.35	195.71^n.s.^ ± 19.55	0.64^∗∗^ ± 0.01
FNFs	224^#^ ± 21.90	120^#^ ± 14.14	254^∗^ ± 19.49	269.79^#^ ± 24.80	0.12^##^ ± 0.01
FNFr	228^#^ ± 22.80	136^n.s.^ ± 35.77	268^n.s.^ ± 45.11	265.24^#^ ± 26.17	0.11^##^ ± 0.01

Values are expressed as mean ± SEM, *n* = 6. ^∗^*p* < 0.05, ^∗∗^*p* < 0.01 versus control group; ^#^*p* < 0.05, ^##^*p* < 0.01 versus ISO group. GP: grape pomace; Fs: fresh; Fr: fermented; FN: Fetească neagră; ISO: isoprenaline; QTc: corrected QT interval; HR: heart rate.

**Table 3:** Serum cardiac marker enzymes.

	AST (UI/L)	ALT (UI/L)	CK-MB (UI/L)
CONTROL	51.36 ± 7.04	47.27 ± 3.17	213.10 ± 46.48
ISO	131.50^∗∗∗^ ± 20.72	113.27^∗∗∗^ ± 13.17	562.50^∗∗∗^ ± 105.20
FNFs	98.52^##^ ± 8.18	86.00^##^ ± 8.14	375.30^##^ ± 33.61
FNFr	103.73^#^ ± 9.34	86.74^##^ ± 5.59	295.20^##^ ± 66.49

Values are expressed as mean ± SEM (*n* = 6). ^∗∗∗^*p* < 0.001 versus control; ^#^*p* < 0.05. ^##^*p* < 0.01 versus ISO group. AST: aspartate transaminase; ALT: alanine transaminase; CK-MB: creatine kinase-MB; Fs: fresh; Fr: fermented; FN: Fetească neagră; ISO: isoprenaline.

**Table 4:** Serum oxidative stress markers.

GP	TOS (*μ*M H_2_O_2_ equiv/L)	TAR (mM trolox equiv/L)	OSI	MDA (nM/L)	SH (mM/L)	NOx (*μ*M/L)
CONTROL	26.92 ± 7.30	1.090 ± 0.001	0.25 ± 0.02	1.09 ± 0.22	0.54 ± 0.06	33.31 ± 4.50
ISO	47.95^∗^^∗^ ± 2.70	1.089^∗^ ± 0.001	0.44^∗∗^ ± 0.02	5.25^∗∗∗^ ± 0.64	0.51^n.s.^ ± 0.10	69.78^∗∗∗^ ± 3.06
FNFs	29.77^##^ ± 4.86	1.091^#^ ± 0.001	0.27^##^ ± 0.04	4.64^#^ ± 0.39	0.50^n.s.^ ± 0.11	65.22^n.s.^ ± 4.04
FNFr	32.53^#^ ± 4.77	1.091^#^ ± 0.001	0.30^##^ ± 0.04	4.46^#^ ± 0.20	0.54^n.s.^ ± 0.07	66.3^n.s.^ ± 3.18

Values are expressed as average ± standard deviation (*n* = 3). ^n.s.^*p* > 0.05, ^∗^*p* < 0.05, ^∗∗^*p* < 0.01, and ^∗∗∗^*p* < 0.001 versus control; ^n.s.^*p* > 0.05, ^#^*p* < 0.05, ^##^*p* < 0.01, and ^###^*p* < 0.01 versus ISO group. GP: grape pomace; Fs: fresh; Fr: fermented; FN: Fetească neagră; ISO: isoprenaline; TOS: total oxidative status; TAR: total antioxidant reactivity; OSI: oxidative stress index; MDA: malondialdehyde; SH: total thiols; NOx: nitrites and nitrates.
